# A Leap Beyond Traditional Methods: A Case Report on the Horizontal Loop Distal Shoe

**DOI:** 10.7759/cureus.59724

**Published:** 2024-05-06

**Authors:** Aditi Gupta, Brahmananda Dutta, Kanika S Dhull, Indira MD, Bhushan Wandile

**Affiliations:** 1 Pediatric Dentistry, Kalinga Institute of Dental Sciences, Kalinga Institute of Industrial Technology (KIIT) Deemed to be University, Bhubaneswar, IND; 2 Pedodontics and Preventive Dentistry, Kalinga Institute of Dental Sciences, Kalinga Institute of Industrial Technology (KIIT) Deemed to be University, Bhubaneswar, IND; 3 Pedodontics and Preventive Dentistry, Jagadguru Sri Shivarathreeshwara (JSS) Dental College and Hospital, Mysore, Mysore, IND; 4 Pediatrics, Jawaharlal Nehru Medical College, Datta Meghe Institute of Medical Sciences, Wardha, IND

**Keywords:** tooth eruption, primary dentition, horizontal loops, pediatric dentistry, space maintainer, distal shoe

## Abstract

This case report presents the successful management of a four-year-old male patient with pain in the lower right back tooth region. Clinical and radiographic examinations revealed the necessity for extraction of tooth 85 due to persistent infection and bone resorption, necessitating space maintenance. A modified distal shoe space maintainer, incorporating horizontal loops for enhanced adjustability, was utilized postextraction. The appliance was fabricated, cemented, and monitored through follow-up visits. At the 24-month recall, the permanent mandibular first molar (tooth 46) was clinically visible, indicating successful space maintenance. Incorporating horizontal loops into the distal shoe space maintainer represents an innovative approach in pediatric dentistry, offering clinicians a versatile tool for managing space loss and promoting optimal eruptive patterns.

## Introduction

Primary dentition plays a crucial role in the maintenance of arch integrity and the development of occlusion in children. Premature loss of primary teeth due to caries, trauma, or other pathologies can lead to various complications, such as space loss, malocclusion, and functional disturbances [1]. To prevent such sequelae, space maintenance becomes imperative, especially in cases where the permanent successor teeth are not ready for eruption [2]. The distal shoe space maintainer is a commonly employed appliance in pediatric dentistry to preserve space following the premature loss of primary molars in the mandibular arch [3]. Traditional designs of the distal shoe utilize a vertical projection extending into the intra-alveolar space, providing support for the developing permanent molars [4]. However, challenges may arise during cementation due to the need for precise adjustment to ensure proper fit and passive contact with the adjacent teeth [5].

Innovations in appliance design aim to address these challenges and improve the efficacy of space maintainers. One such innovation involves the incorporation of horizontal loops into the conventional distal shoe design. Horizontal loops offer increased flexibility and adjustability, allowing for more accessible adaptation and cementation of the appliance [6]. This modification enhances the clinician's ability to achieve optimal space maintenance while minimizing the risk of occlusal interferences and malocclusion [7]. This case report presents a novel approach to space maintenance utilizing the horizontal loop distal shoe, highlighting its advantages in terms of fabrication, cementation, and clinical outcomes. By incorporating horizontal loops into the design, clinicians can effectively manage space loss in primary dentition, promoting the proper eruption of permanent teeth and minimizing the need for orthodontic intervention.

## Case presentation

**Figure 1:**
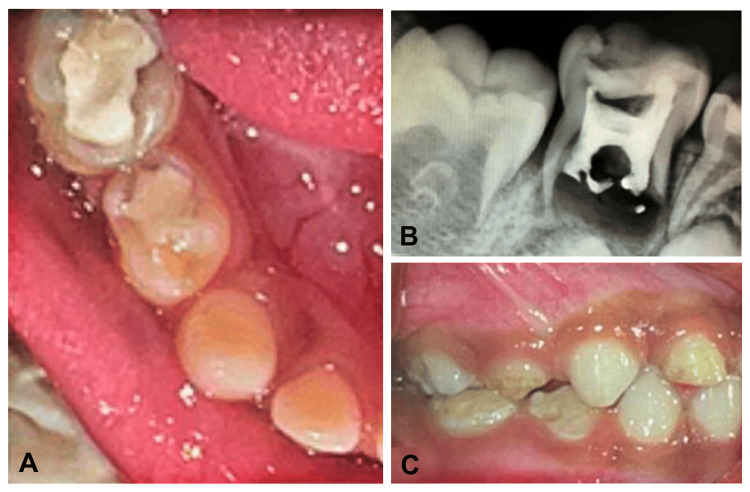
A) Preoperative occlusal view, B) preoperative radiograph, C) preoperative occlusion (right side)

A four-year-old male patient was referred to the Department of Pediatric and Preventive Dentistry with a chief complaint of experiencing pain in the lower right posterior tooth region, persisting for one week. The patient exhibited a state of good nutrition and coordination, displaying a positive assessment according to Frankl's behavior scale. Clinical examination revealed the presence of dental restorations associated with teeth 84 and 85 (Figure 1A-1C). The radiographic evaluation indicated radiopacity within the root canals of tooth 85, suggestive of obturating material. Additionally, a furcal radiolucency accompanied by root resorption was observed, indicative of persistent infection and bone resorption, thus implying a guarded prognosis (Figure 1B). Tooth development stages were assessed, revealing Nolla's stage 4 for tooth 45 and stage 6 for tooth 46.

The proposed treatment plan involved the extraction of tooth 85 followed by implementing a modified distal shoe space maintainer. Horizontal loops were incorporated into the conventional design to facilitate adjustments during the cementation process. The patient's guardian was thoroughly briefed on the treatment protocol and provided consent.

**Figure 2:**
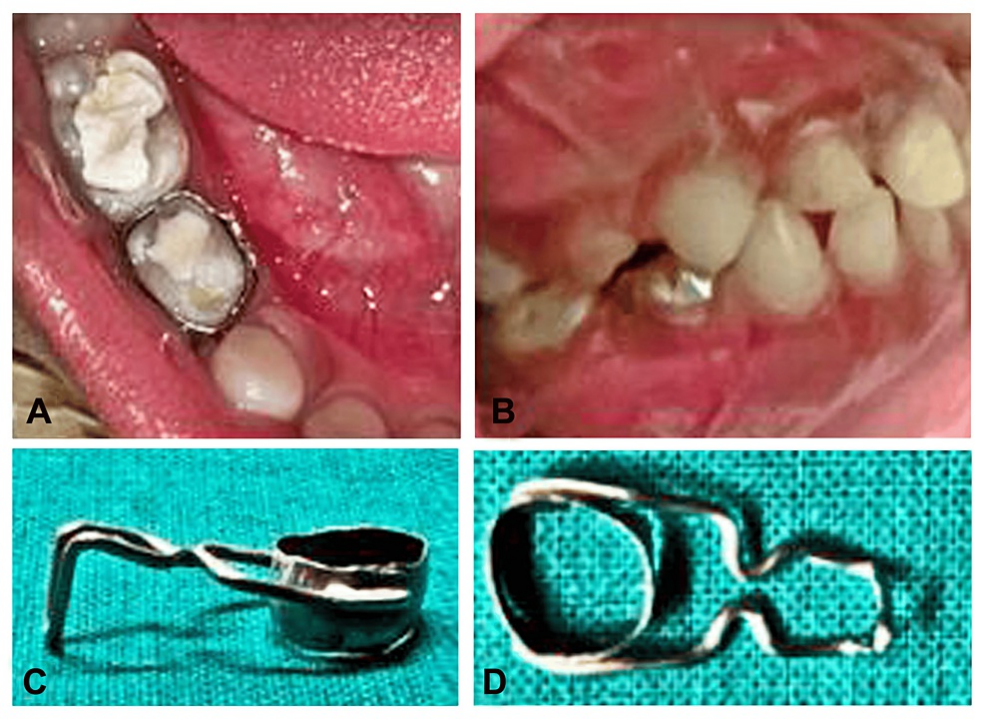
A) Banding i.r.t 84 (occlusal view), B) banding i.r.t 84 (right side occlusion), C-D) fabricated and soldered horizontal loop distal shoe

Band adaptation was conducted for tooth 84 during the initial visit, followed by the acquisition of alginate impression records and band transfer. Subsequently, the band was secured, and working models were created employing a dental stone (Figure 2A-2B). Utilizing the primary second molar as a reference, the extension of the distal shoe was estimated, and the vertical depth of the intra-alveolar projection was determined radiographically. A buccolingual deep groove was fashioned in the cast. The wire component, crafted from 21-gauge stainless steel wire, featured a V-shaped vertical projection transitioning into buccal and lingual arms, incorporating horizontal loops and terminating with close adaptation to tooth 84. The ends of the wire component were soldered buccally and lingually to the band employing a silver solder. The completed appliance underwent retrieval, followed by meticulous finishing and polishing to eliminate sharp edges and excess material (Figure 2C-2D).

**Figure 3:**
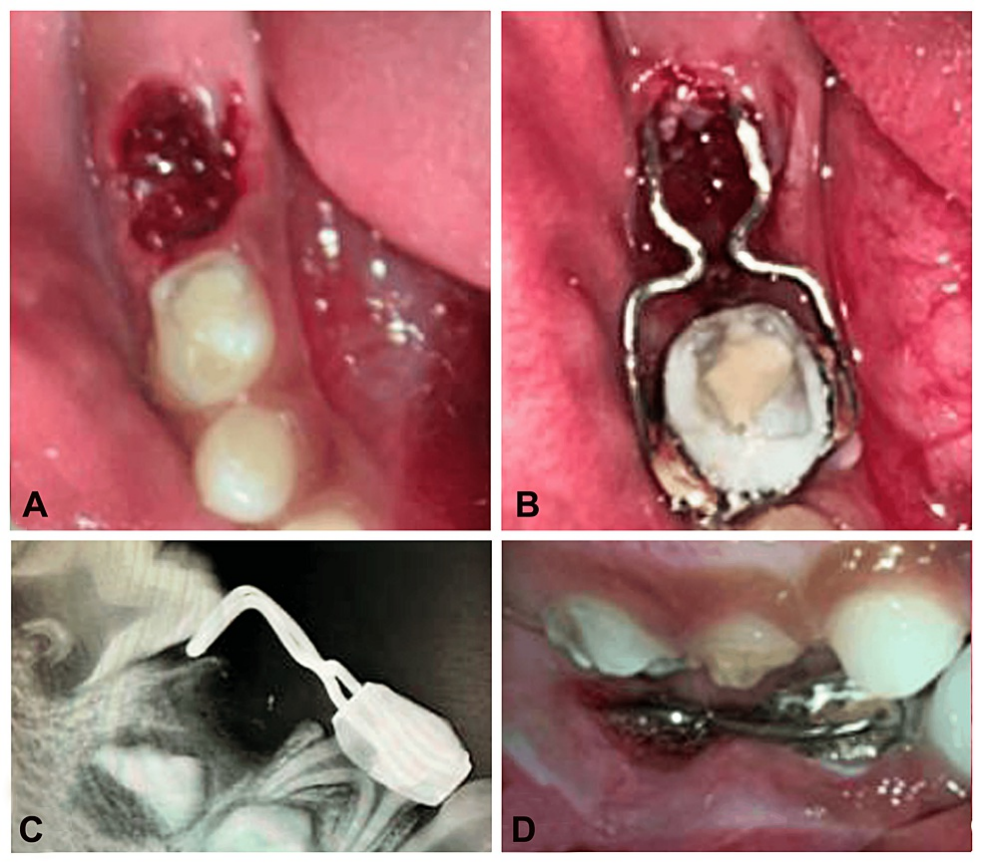
A) Tooth socket post-extraction i.r.t. 85, B) post-cementation occlusal view, C) radiographic confirmation of the position i.r.t. 46, D) trial of horizontal loop distal shoe (right side occlusion)

**Figure 4:**
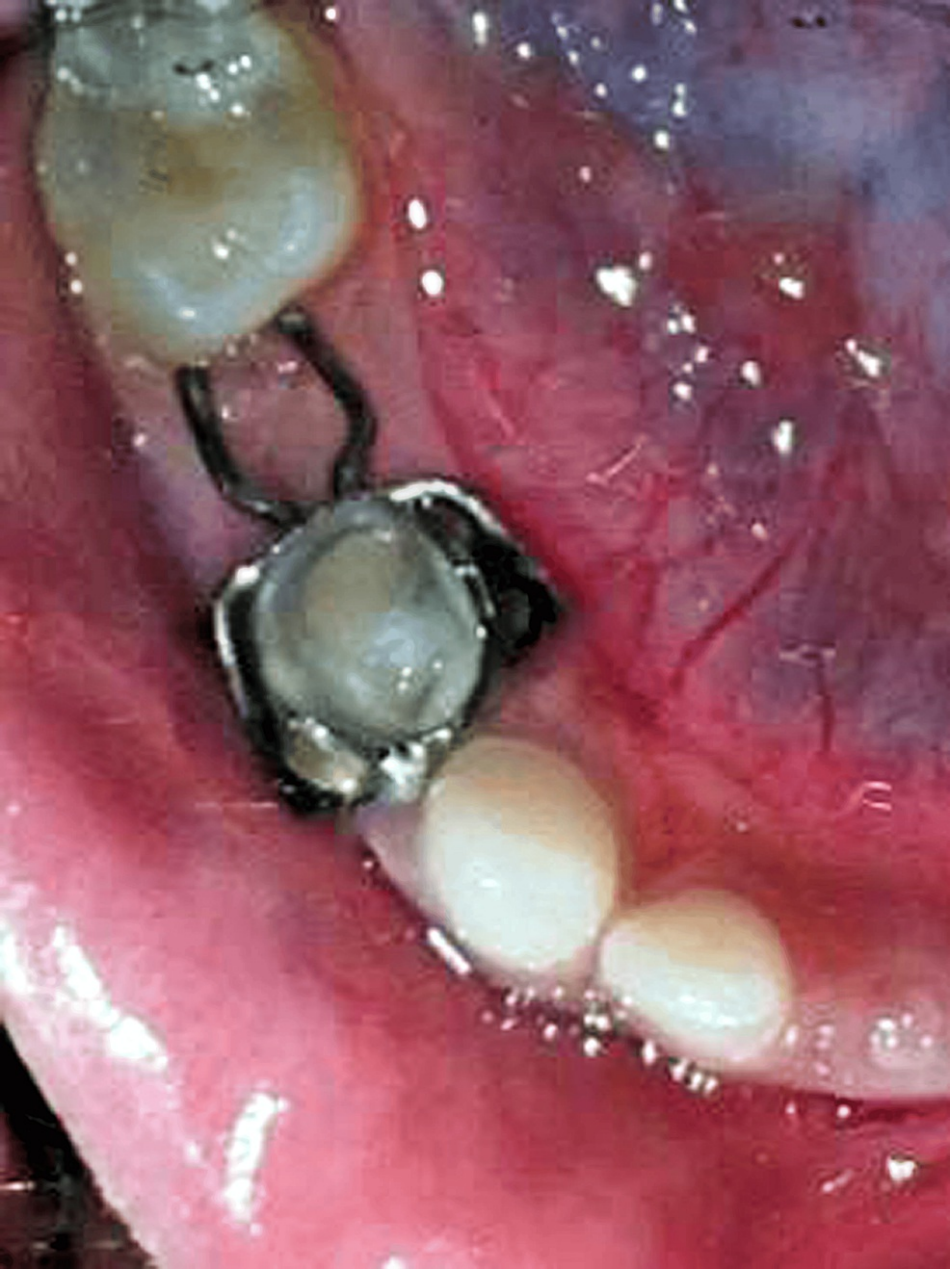
The follow-up at 24 months

Following the extraction of tooth 85 under antibiotic coverage, hemostasis was achieved by applying a pressure pack (Figure 3A). Subsequently, the appliance was delicately inserted, first positioning the band followed by the intra-alveolar projection. Radiographic confirmation of passive contact between the mesial marginal ridge of tooth 46 and the appliance was verified. Adjustments in the anteroposterior direction were executed with the aid of horizontal loops. The final appliance was cemented utilizing a luting agent (GC Gold Label Fuji Type 1), and a radiograph was obtained (Figure 3B-3D). The patient and guardians received detailed instructions on oral hygiene practices, including regular mouth rinsing with povidine-iodine (Betadine) mouthwash post-meals and application of a remineralizing agent (GC Tooth Mousse Plus®). A follow-up appointment was scheduled every three months to maintain optimal dental health. At the 24-month follow-up, the permanent mandibular first molar (tooth 46) was clinically evident (Figure 4). However, due to the partial eruption of tooth 46, it was planned to replace the horizontally looped distal shoe with a reverse band and loop space maintainer.

## Discussion

Managing space loss in pediatric dentistry is crucial to prevent malocclusion and facilitate the proper eruption of permanent dentition. The case presented highlights the successful utilization of a modified distal shoe space maintainer with horizontal loops for effective space maintenance following the extraction of a primary molar. The distal shoe space maintainer, initially described by Nance in 1947, is a commonly used appliance for maintaining space following the premature loss of primary molars [8]. Traditionally, the distal shoe incorporates a vertical intra-alveolar projection to guide the eruption of the permanent first molar. However, adding horizontal loops in the present case provides several advantages. By incorporating horizontal loops into the design, the appliance offers enhanced adjustability during cementation, allowing for precise positioning to ensure proper contact with the mesial marginal ridge of the adjacent tooth [7]. This adjustability is particularly beneficial in cases where there is variability in the eruption patterns of permanent teeth, as seen in the partial eruption of tooth 46 in this case.

Moreover, the horizontal loops allow for improved flexibility and adaptation to the contours of the oral cavity, reducing the risk of appliance dislodgement and enhancing patient comfort [9]. The ability to adjust in the anteroposterior direction ensures optimal alignment and contact between the appliance and the adjacent teeth, promoting favorable eruptive forces and minimizing the risk of occlusal disturbances [10]. Incorporating horizontal loops into the distal shoe space maintainer represents a leap beyond traditional methods, offering clinicians a versatile and effective tool for managing space loss in pediatric patients. By providing greater flexibility and adjustability, this innovative approach addresses the limitations of conventional designs and enhances the predictability of treatment outcomes. In addition to its clinical implications, the case underscores the importance of comprehensive oral health education and regular follow-up visits. Proper oral hygiene measures, including regular mouth rinsing with antimicrobial mouthwash and application of remineralizing agents, are essential for maintaining the health of the dentition and preventing secondary caries and periodontal complications [11]. Furthermore, scheduled recall appointments allow for timely monitoring of eruption patterns and appliance adjustment as needed to accommodate changes in the dentition.

## Conclusions

In conclusion, utilizing a modified distal shoe space maintainer with horizontal loops represents a significant advancement in managing space loss in pediatric dentistry. By addressing the limitations of traditional designs, incorporating horizontal loops enhances the predictability of treatment outcomes while minimizing the risk of occlusal disturbances and appliance dislodgement. Moreover, the successful outcome observed in this case underscores the importance of comprehensive oral health education and regular follow-up visits in maintaining the health of the dentition and preventing secondary complications. Further research and clinical studies are warranted to validate this novel technique's long-term efficacy and benefits, ultimately improving the quality of care for pediatric patients in need of space maintenance interventions.
